# Localizing potentially active post-transcriptional regulations in the Ewing's sarcoma gene regulatory network

**DOI:** 10.1186/1752-0509-4-146

**Published:** 2010-11-02

**Authors:** Tatiana Baumuratova, Didier Surdez, Bernard Delyon, Gautier Stoll, Olivier Delattre, Ovidiu Radulescu, Anne Siegel

**Affiliations:** 1 Systems Biology Group, Life Science Research Unit, University of Luxembourg,162A Avenue de la Faiencerie, Luxembourg, L-1511, Luxembourg; 2 Work done at: IRMAR, Université de Rennes 1, Rennes, France; 3 UMR 6625, CNRS, Rennes, France; 4 Genetics and Biology of Cancers, Institut Curie, Paris, France; 5 Unité 830, INSERM, Paris, France; 6 Service bioinformatique, Institut Curie, Paris, France; 7 Unité 900, INSERM, Paris, France; 8 Service bioinformatique, Mines ParisTech, Fontainebleau, France; 9 DIMNP, Université de Montpellier 2, Montpellier, France; 10 UMR 5235, CNRS, Montpellier, France; 11 Symbiose project team, INRIA, Rennes, France; 12 UMR 6074, CNRS, Rennes, France; 13 IRISA, Université de Rennes 1, Rennes, France

## Abstract

**Background:**

A wide range of techniques is now available for analyzing regulatory networks. Nonetheless, most of these techniques fail to interpret large-scale transcriptional data at the post-translational level.

**Results:**

We address the question of using large-scale transcriptomic observation of a system perturbation to analyze a regulatory network which contained several types of interactions - transcriptional and post-translational. Our method consisted of post-processing the outputs of an open-source tool named BioQuali - an automatic constraint-based analysis mimicking biologist's local reasoning on a large scale. The post-processing relied on differences in the behavior of the transcriptional and post-translational levels in the network. As a case study, we analyzed a network representation of the genes and proteins controlled by an oncogene in the context of Ewing's sarcoma. The analysis allowed us to pinpoint active interactions specific to this cancer. We also identified the parts of the network which were incomplete and should be submitted for further investigation.

**Conclusions:**

The proposed approach is effective for the qualitative analysis of cancer networks. It allows the integrative use of experimental data of various types in order to identify the specific information that should be considered a priority in the initial - and possibly very large - experimental dataset. Iteratively, new dataset can be introduced into the analysis to improve the network representation and make it more specific.

## Background

### Network modeling and data analysis in Cancer Systems Biology

During the last decade, interest in using network models for elucidating mechanisms of disease has constantly increased [1]. In particular, identifying the pathways that are responsible for malignancy is an important challenge in Cancer Systems Biology [2,3]. Although it is now accepted that cancer is a genetic disease, the levels of gene expression remain, for many reasons, unreliable indicators of causation [4]. First, the genetic perturbations produce a multitude of changes, not all related to the phenotype. Second, the mutated genes initiating the processes are not detectable as differentially expressed. Last but not least, important modifications of the pathways result from post-translational interactions that are independent of the changes at the mRNA level.

Information on protein-protein and protein-DNA interactions has recently become available for human interaction pathways. Many groups combine literature information and machine learning to build network models for disease. It has been proposed that networks can be used as filters to identify genes implicated in cancer. For instance, Chuang and colleagues used network models to improve markers for tumor classifications [3]. They identified mutated genes in cancer from their effect on connected sub-networks of differentially expressed genes. The sub-networks are proposed as classifiers of tumors and could also serve to generate new hypotheses about the disease. A similar idea has been investigated by Ergun and colleagues [2]. They identified groups of genes whose expressions are most affected by disease. In order to find these genes, the dynamics of the network is modeled by simplified differential equations. The disease is supposed to affect transcription rates by multiplying them by a gene-dependent factor. Estimating this factor from data allows one to rank genes according to a z-score representing the influence of the disease. Another method for determining the genes most affected by disease has been applied to cancer by Mani and colleagues [5]. Mutual information (MI) quantifies the degree of dependence between interacting genes. By computing the change of MI induced by various tumor phenotypes in cohorts of patients, one can assign to each tumor phenotype a set of genes that are most affected. Various network-based classifiers of tumors have been proposed elsewhere [6,7].

### Constraint-based approaches for hypothesis generation

All approaches mentioned above face difficulties in the quality of the network representation. Indeed, in any network-based study of disease, the first step is the network construction. "Gold standard" evidence from curated databases and from the literature allow integrating a large amount of experimental and computational evidence. Such evidence is gathered in a model, often represented by an interaction graph that is prone to incompleteness and uncertainty. Mutual information [5] or other machine-learning methods [2,3] can be used to fill in gaps in the network or, alternatively, to discard interactions if their presence is not supported by data. Nonetheless, the logical implications of the confrontation between network models and data are insufficiently explored by these methods.

A different class of approaches, less developed in cancer studies, uses model-checking and constraint-based analysis to test and exploit the logical consistency between model and data. Several types of queries can be performed. They can be dynamic, although using different temporal logics, like those implemented in the Biocham [8], BioNetGen [9] or GeneNetAnalyser [10] softwares. Queries may also be static, particularly in the case of middle and large-scale networks. For instance, Bowers and colleagues rely on static logic relationships to investigate protein network organization [11]. Baumbach and colleagues also used static rules to perform predictions on corynebacterial regulatory networks [12]. We have recently designed a tool for constraint-based analysis of interaction networks [13] named BioQuali which aims at automatizing such approaches. The tool solves large systems of qualitative equations which connect the variations of a node to the variations of its precursors in the interaction graph. Applied to network models, BioQuali can validate the existence of interactions in a network and predict the variations of nodes that are not directly measured. In case of conflict between model and data, the origin of the conflict is localized by analyzing the propagation of the constraints. Then, new experiments can be suggested to check the new hypotheses [14]. In this context, constraint-based analysis complements statistical approaches in hypothesis generation.

### Alternative network representation coping with realistic interactions and data

The main difficulty faced when applying such formal methods lies in the network representation. Indeed, most of models studied in this context are built from curated databases or the literature. According to the level of detail in the knowledge and the size of networks at hand, most models do not discriminate between mRNA and protein levels. The variables (node attributes) are mRNA levels observed with microarrays. However, protein levels are not always correlated to mRNA levels, especially in cancer systems. In such situations, it is vital to distinguish between transcriptional, post-transcriptional and post-translational interactions. Furthermore, new types of nodes should be used whose attributes, though constrained by the network, are not available in the experiment. Predicting the attributes of these nodes (for instance, protein activities) is essential for assessing the effectiveness of the interactions.

In this paper we propose a constraint-based analysis of a cancer network model. Our main concern is to identify and account first for uncorrelated protein and mRNA levels, second for post-translational interactions. We automatically modify the network representation so that each gene satisfying our given criteria is represented by two nodes, an mRNA and a protein node. Using the BioQuali tool, we check consistency between the data and two descriptions of the system: the initial network built from a curated database and the modified network with distinct interaction levels. We investigate the results of the constraint-based analysis tool to predict protein activities and identify *active *regulations in the network. Within this framework, the notion of activity is understood as interactions in the network that are logically required to obtain a given set of observations. Lastly, we confront the results of our analysis to new data in order to refine the model.

Other network representation studies, such as those performed by Mani and colleagues [5], have suggested distinctions between types of proteins - transcription factors, non-TF and modulators according to their function in the cell. However, the representation of network interactions and their relation to data is not equivalent to ours. The network model in our constraint-based approach is a signed interaction graph. The information on the sign of interactions (inhibition, activation) is essential for our analysis. MI-based networks use oriented interactions for which the sign information is not important. More importantly, in these models post-translational modifications are represented as modulations of the interactions between transcription factors and their targets. Our decision to separate protein and mRNA nodes allows a more rigorous analysis of the constraints. It can exploit data of both mRNA and protein types, and therefore performs hypothesis generation.

### Analysis of a cancer network

To test our method, we used it to study Ewing's sarcoma. Ewing's sarcoma is the second most common malignant bone tumor in children and young adults. Delattre and colleagues showed that it is associated in more than 80% of cases with the t(11;22)(q24;q12) chromosomal translocation [15,16]. The latter induces the expression of the chimeric protein composed of the N terminal part of the EWS gene with the ETS family member FLI1 c-terminal part [17]. Consequently, EWS-FLI acts as an aberrant transcriptional activator/repressor in Ewing's sarcoma by altering the expression of specific target genes [18,19]. Our decision to analyze Ewing's sarcoma is motivated by two aspects. First, the key genetic perturbation of the Ewing network is known to be EWS-FLI1, a transcription factor present in the majority of Ewing's tumors [20]. Second, large datasets accumulated on this cancer are available, making it of particular interest for our approach. In cell lines, the changes of genetic program induced by siRNA inhibition of the oncogene lead to cell cycle arrest in G0/G1. Upon reactivation, most of the expression levels change and the phenotypes are reversed. Altogether, this provides quite reliable datasets for the study of the network perturbation. However, the precise regulatory pathways of the EWS-FLI1 oncogene are not yet fully elucidated. Identifying gene interactions that are potentially involved in the regulation of EWS-FLI1-related pathways are of great interest for studying Ewing's sarcoma.

## Methods

### Gene expression data, Ews-Fli1 regulatory network

#### Data on gene expression upon Ews-Fli1 knock down/rescue in Ewing's cancer cells

Transcriptome time-series data were obtained using Affymetrix U133A microarray as published in [21]. In this paper, Ewing cells were profiled upon EWS-FLI1 knocked down (with retroviral-mediated RNAi EF-2-RNAi construct) and then rescued based on a tetracycline-inducible EWS-FLI1 cDNA. Predicted variations of gene expression during inhibition of EWS-FLI1 were validated with additional experiments as follows. For RT-QPCR and Western blotting experiments, EWS-FLI1 transcript was silenced and reactivated using a tetracycline inducible shEWS-FLI1 specific construct in clones derived from the A673 cell lines. Levels of IGF2 mRNA (taqman Hs01005963_m1, Applied biosystems) and FASLG mRNA (forward primer: ggaaagtggcccatttaaca reverse primer: ccagaaagcaggacaattcc) were measured by real-time quantitative polymerase-chain reaction (RT-QPCR, normalized to RPLP0). Western blotting was performed with IGF2 (AB9574, Abcam) and FASLG (AB15285, Abcam) antibodies.

#### Regulatory network of EWS-FLI1 chimeric oncogene

An annotated gene regulatory and signaling model was designed by our colleagues from Institut Curie [22]. The model involves 130 genes selected according to the strength of their response on the inhibition/reactivation of EWS-FLI1. Using information from BIOBASE [23] and manual curation of the literature, around 300 interactions were selected to describe signaling pathways that regulate key functions involved in tumor progression (cell cycle phase transitions, apoptosis and cell migration). Products in the networks correspond to genes, proteins, phosphorylated proteins - such as RB1_phosphorylated - and protein complexes such as the complex made of CCNB1 and CDK2. Due to lack of precise knowledge, additional nodes were added to describe the effects of families of proteins - such as the RAC family which includes RAC1, RAC2 and RAC3. Interactions issued or targeting such a node gather all interactions known for at least one element in the family. Finally, the network included some nodes to describe the phenotypic effects of the oncogene - apoptosis, cell migration, cell cycle anaphase, -G2, -M and -S phases.

From this annotation of the network we have extracted an *interaction graph *as follows. Nodes of the graph are given by the products that appear in the initial model. Every interaction in the initial annotated model is mapped to a labeled edge of the interaction graph. Every edge represents a positive ('+' meaning up-regulation), negative ('-', down-regulation) or dual ('?', the actual regulation is unknown) influence of a source node (precursor) on a target node (successor) (see Additional file 1: Interaction graph adapted from the original network). Each interaction is also annotated with respect to the type of interaction (transcriptional or post-translational, not shown). The interaction graph was further modified according to the method proposed below to enhance transcriptional and post-translational effects (see the forthcoming section "First add-on: changing the descriptive level of the network" and Additional File 2: Modified interaction graph).

#### Extracting average trend of gene response to the oncogene inhibition

We used a dedicated gene-level summarization and data filtering process to analyze the Affymetrix data published in [21]. Our goal was to capture the average trend of the response of the network nodes to oncogene inhibition, that is, to identify nodes which are either correlated or anti-correlated to the oncogene inhibition expression pattern. A pre-normalization procedure was performed in [21]: (A) Checking for mis-targeted and non-specific hybridization; (B) Background subtraction, in order to remove the fluorescence induced by factors other than hybridization of RNA to the chip. (C) Normalizing the data using a Robust Multiarray Average (RMA) method to remove systematic bias such as platform-specific variations or the influence of non-biological factors.

Starting from these data, a gene-level summarization was performed by grouping the probe sets corresponding to the same gene. Within such groups, all probe set lines were kept instead of substituting them with the single mean or median value. This allowed capturing the statistical significance of the gene response including all its transcript variances. Such gene groups of the full microarray were then filtered to select only those genes relevant for the network. An outlier removal procedure was applied to the selected gene-level grouped data. Data points were considered as outliers if their residuals were larger than two standard deviations (SD) of the gene dataset. The residuals were computed as the distance from the regression line built for each gene to each of the gene set data points.

Finally, genes were arranged according to the significance of their responses to the oncogene inhibition in the Affymetrix microarrays [21]. To that end, the response curves of the transcripts corresponding to a gene were approximated with a linear function using linear regression. A gene was considered as significantly responding to the oncogene inhibition if the regression line fitted its response curves with a statistical significance level of less than 5% and if the slope of the regression line significantly differed from zero. The latter condition was checked indirectly using a two-sample location test (Student's t-test), which was performed on the initial and final points of the time series. The significance threshold was again fixed at 5%. Gene variation signs were provided either by signs of slopes (for linear regression) or by signs of variations (for Student's t-test), given that both parameters are statistically significant (p-value less than 5%). A final adjustment to multiple pair-wise tests - Holm-Bonferroni method, family-wise error rate, false discovery rate - was not considered as relevant since the list of genes had been previously reduced to a much smaller one, corresponding to the studied network.

### Constraint-based analysis: automatic reasoning tool and its add-ons

#### BioQuali tool

We used an open-source software tool named BioQuali to perform the qualitative analysis of EWS-FLI1 regulatory network [13]. BioQuali assesses the compatibility between the network topology and the expression variations induced by a disturbance of a system. It performs automatic reasoning by propagating observations along the network - as usually done when reasoning about regulations - and checking whether this propagation either yields a contradiction or generates new deductions. The tool is publicly available at http://genoweb1.irisa.fr/Serveur-GPO/outils/interactionNetwork/BIOQUALI/[13]

The propagation of information is modeled by establishing a set of rules connecting the sign of a node variation to the signs of its precursors' variations in the network. The main rule used in this paper was as follows: "the sign of a variation of a node cannot be opposite to the sign of variations of all the influences it gathers from its precursors in the network". This rule was mathematically proven to be valid if the initial and the final states of the network are steady states of the systems [24]. Note however that stronger rules may be used when precise knowledge on interaction is available [14]. The full set of rules is encoded as a system of qualitative constraints over its inputs (interaction graph and observations on node variations). It may therefore be considered as a constraint-based modeling tool. Solving such a system is computationally difficult. The BioQuali tool uses a dedicated constraints solver based on a decision diagram to overcome the computational difficulty and solve systems of biological constraints in a reasonable time. More details may be found in the software publication [13,25].

**Figure 1:**
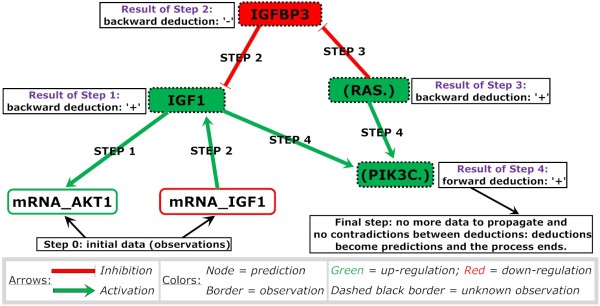
**BioQuali reasoning: logical steps leading to predictions**. The tool encodes knowledge and observations into a system of qualitative equations which is solved with an efficient algorithm [25]. The algorithm generates the complete set of solutions of these equations and identifies invariants of the set of solutions, that is, node values that are constant throughout the entire set of solutions. These invariants are called predictions. They can also result from the logical deduction process detailed above. The tool computes predictions without detailing the reasoning steps, which would be impossible for large scale systems. Step 1 (backward deduction). The node mRNA AKT1 is observed as up-regulated. It is regulated by IGF1 only. Therefore IGF1 should be up-regulated to explain the observation. Step 2 (backward deduction). IGF1 increase cannot be derived from an increase of its transcriptional activity since its mRNA is down-regulated. The only possible explanation is a decrease of its inhibitor IGFBP3. Step 3 (backward deduction). Consequently, the only regulator of IGFBP3 should be up-regulated. Step 4 (forward deduction). All incoming regulations on PIK3C tend to increase it. This should result in an increase of the node activity. Final step. No additional input or former deduction propagates. Deductions are all in agreement with each other. They are denoted as predictions and the process ends. Alternatively, if a decrease of PIK3C is observed or deduced, the full process would fail, all deductions would be discarded and an inconsistency diagnosis would be generated.

The complete system of rules is analyzed to decide whether the interaction graph is compatible with the input data. Consistency means first that all the interactions in the graph are in logical agreement with each other and, second, that they do not conflict with experimental observations. In case of disagreement, BioQuali points out the inconsistent parts of the interaction graph. In case of consistency, BioQuali generates a set of predictions. They correspond to variations of non-observed nodes that can be deduced from the observed variations - that is, available data - by applying the generic biological rule introduced earlier. In Figure 1, we detail a deduction process leading to such predictions. Notice however that the BioQuali tool does not explicitly compute all the steps in this process: the software encodes the steps into equations and then solves the resulting system with efficient methods [25].

**Figure 2:**
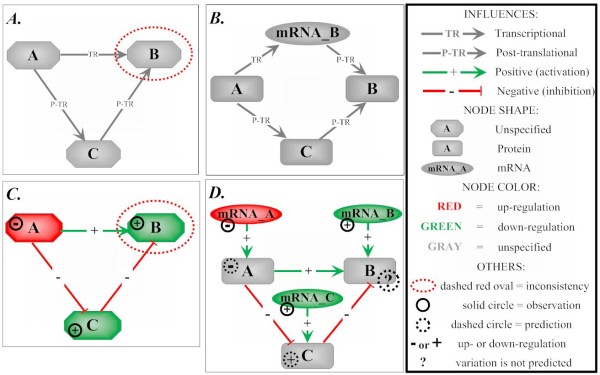
**Types of inconsistencies caused by undifferentiated mRNA and proteins in the network representation**. On both images *A *and *C*, inconsistencies occur on the node 'B'. The first example (*A*) is inconsistent because the product of gene expression, represented by node 'B' is a target of both transcriptional (TR) and post-translational (P-TR) regulations, which is not possible since transcriptional interactions should target mRNAs, while post-translational interactions target proteins. This inconsistency is removed by the differentiation between mRNAs (node 'mRNA_B') and proteins (node 'B') as shown on image *B*. The second type of inconsistency (*C*) is caused by the association of experimental data on variation in mRNA with a node which should represent a protein because it is a target of post-translational regulations. This inconsistency can also be avoided if mRNAs and proteins are distinguished in the network (*D*).

Two other examples of predictions are shown in Figure 2. They may be classified into two types with respect to the constraint propagation as follows:

*• Forward predictions *are those which are straightforwardly deduced from precursors of a predicted node. Such predictions reflect the consequence of the concerted action of the precursors on a predicted node in the network. If the actions of all the precursors are known to have the same sign, the node will potentially vary in the direction that is imposed by this concerted action of its precursors.

*• Backward predictions *are deduced from the successors of a predicted node. These predictions represent the only possible explanations of the associated experimental observations and (or) the topology of the network model. In contrast to forward ones, backward predictions do not reflect the smooth propagation of an interaction along the network. They may rather be seen as the result of an automatic global reasoning to fill in gaps between observations on node variations, in order to find the only possible reasons for such observations.

Sorting predictions according to their significance can be performed in different ways. A statistical significance may be computed by pointing out those predictions that are very specific to the dataset - slightly changed dataset will no more generate a prediction. This approach was used in [14] to test the robustness of a network and the validity of reasoning rules. An alternative is to consider all the predictions if they are not too numerous. As we will detail in the sequel, we will consider all predictions and favor those providing information on post-translational processes.

#### First add-on: changing the descriptive level of the network

The initial network represents a summary of our biological knowledge about the pathways putatively influenced by the oncogene. The nodes of this network represent proteins, family of proteins, protein complexes, and phenotypes. We have modified the network in order to obtain an interaction graph, i.e. an oriented signed graph whose arrows (directed edges) represent interactions. An arrow connects two nodes if the tail node influences the production of the head node. We used the following criteria to modify the node representation of the initial network according to the transcriptional or post-translational nature of interactions. This allowed us to point out predictions which are relevant from the post-translational viewpoint.

• Whenever possible, families of proteins are split into representatives.

• For each protein which is a target of at least one transcriptional regulation or which is coded by a gene observed at the transcriptional level, the modified network will contain two nodes: the mRNA and the protein. Alternative splicing can be taken into account at this level, because several proteins can be coded by the same gene and thus emerge from the same mRNA.

• The mRNA nodes are targets of all transcriptional regulations and up-regulate protein nodes - see Figure 2 for details. The underlying assumption here is that increasing (or decreasing) the production of an mRNA tends to increase (or decrease) the production of the corresponding protein.

• The other protein nodes (not targets of post-translational interactions or whose mRNA are not observed) remain as such (do not generate an mRNA node).

• In the interaction graph, the precursors of protein complexes are the constituent proteins.

• All protein-protein interactions are designated as post-translational.

#### Second add-on: post-processing of BioQuali results and classification of predictions

The post-processing of BioQuali predictions was designed to enhance information on the activity of post-translational interactions. We pointed out those predictions which contradict the naive dogma of a correlation between mRNA and protein levels.

• Type I. Predicted protein variations opposing mRNA variations. Assuming that the variation of a protein is correlated to the variation of its mRNA unless it is perturbed by a post-translation process, a prediction of Type I suggests that a post-transcriptional regulation in the network strongly reverses the transcriptional production of the predicted protein. Type I predictions are usually obtained as backward deductions.

• Type II. Predicted protein variations with non-significant mRNA variations. Predictions of this type are similar to predictions of Type I and suggest the existence of active post-translation interaction. However, the argument in favor of active interaction is weaker here than for Type I predictions. Indeed, a non-significant mRNA variation can be due to an inaccuracy in the dataset - such as a lack of sensitivity of the microarray technique used to detect the variation of the predicted gene.

• Type III. Predicted protein variations correlated to mRNA variations. This type of predictions is frequently derived from forward deductions, especially when the observed mRNA is the only precursor of the predicted protein. Such predictions simply suggest that the transcriptional interactions are dominating.

This classification is obtained by performing a complete comparison of predictions about mRNA and protein nodes. This functionality will be included in a forthcoming distribution of BioQuali software.

## Results and Discussion

### Identification of significantly responsive genes

In the BioQuali setting, we call "observation" a gene with significant variation. The set of observations used for the qualitative analysis of the tumor oncogene network was inferred from publicly available transcriptome time series data from Smith and colleagues [21], see Methods section. Time series provide the transient, that is, intermediate values of the variables between the initial and final states. Nonetheless, the BioQuali formalism is not concerned with the details of the transients, but only with the average trend of the response: increase or decrease between the two states.

Such an average trend has a meaning also for variables whose level in individual cells oscillates, as for instance genes and proteins controlling the cell cycle. For these variables, the average trend characterizes the change in the population of cells (in our case the inhibition of the oncogene induces a stop of proliferation in G1 and up-regulates G1 specific genes). In both cases, a linear regression method was applied (see Methods section) as the simplest approximation which captures the average trend of the response.

The analysis of the microarray time series data is summarized in Table 1 and in Additional file 3: Results of statistical analysis of microarray data [21]). It contains 33 genes of the network significantly responsive on EWS-FLI1 inhibition.

**Table 1:** Significantly varying products upon EWS-FLI1 oncogene silencing

**Node name**	**Variation**	**Node name**	**Variation**	**Node name**	**Variation**
mRNA_RAS	+	mRNA_ECM1	+	mRNA_RBL2	-
mRNA_CCNE_	+	mRNA_ECM2	+	mRNA_SKP2	+
mRNA_NFKB_	+	mRNA_FAS	-	mRNA_SOS2	+
mRNA_PIK3C_	+	mRNA_FASLG	+	mRNA_TNFAIP3	+
mRNA_PIK3R_	-	mRNA_IER3	+	mRNA_TNFRSF1A	+
				
mRNA_RAC_	+	mRNA_IGF1	-	Phenotypic_observations	
				
mRNA_RHO_	+	mRNA_IGFBP3	+		
mRNA_TGFB_	+	mRNA_JUN	+	EWS-FLI1	-
mRNA_TNF_	+	mRNA_MAPK8	+	Cell_Cycle_Anaphase	-
mRNA_AKT1	+	mRNA_MYC	-	Cell_Cycle_G2	-
mRNA_CDKN1A	+	mRNA_MYCBP	-	Cell_Cycle_M	-
mRNA_CDKN1C	+	mRNA_PDGFRB	+	Cell_Cycle_S	-
mRNA_CYCS	-	mRNA_PRKCB1	-	Cell_Migration	-
mRNA_E2F5	-	mRNA_RASA1	+	Apoptosis	+

List of observations used as an input set for BioQuali. Table contains the list of significantly varying genes resulting from the analysis of microarray data [21]. The last seven lines of the third column contain additional variations on so-called 'phenotypical nodes' and on the EWS-FLI1 protein. They summarize the current literature-derived knowledge on cell behavior upon silencing EWS-FLI1 oncogene.

This set of significant variations was complemented with variations over non-transcriptional products, including the variation of EWS-FLI1 protein and variations of 'phenotypic' nodes (representing apoptosis, cell migration, cell cycle anaphase, -G2, -M and -S phases). These variations were deduced from biological observations (see Additional file 4: Set of experimental observations).

### Consistency analysis of EWS-FLI1 regulatory network

We performed a consistency analysis of the EWS-FLI1 regulatory network two times: first in its original shape deduced from BIOBASE and second time in its modified shape to enhance post-translational processes. As an input dataset to BioQuali we considered the observations resulting from the inactivation of the oncogene performed by Smith and colleagues [21]. Since EWS-FLI1 is down-regulated during this experiment, the variation of EWS-FLI1 was set to '-'. According to the Identification of significantly responding genes performed above, genes and proteins which are anti-correlated to EWS-FLI1 and are up-regulated upon the oncogene silencing have '+' as a sign of variation, while correlated genes and proteins have '-' as a sign of variation.

**Figure 3:**
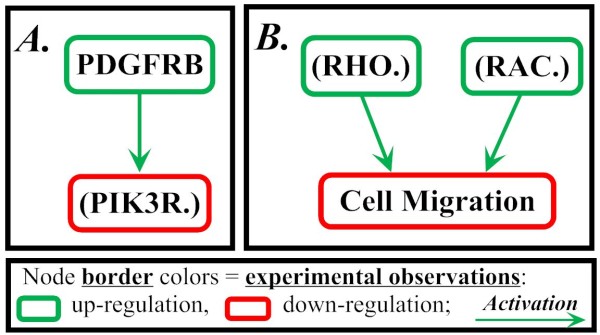
**Inconsistencies in the original interaction graph**. The inconsistencies indicate parts of the network where the original regulatory model fails to satisfy the generic biological rule of BioQuali. In part *A*, node (PIK3R.) has only one precursor, PDGFRB, and its variation (PDGFRB = '+') cannot explain the variation of (PIK3R.) ((PIK3R.) = '-'). In part *B*, the variation of the phenotypic node 'Cell Migration' ('Cell Migration' = '-') cannot be explained by either of the variations of its precursors: (RAC.) = '+' and (RHO.) = '+'.

We first used the BioQuali tool to analyze the interaction network directly inferred from EWS-FLI1 BIOBASE regulatory network, prior to changing its descriptive level: at this stage, mRNA and protein nodes were not separated. The network was compared to the set of observations resulting from transcriptome time series data analysis. The analysis showed that this network with non-distinguished gene products has two inconsistent parts on nodes '(PIK3R.)' and 'Cell Migration' (Figure 3). Both cases of inconsistency resulted from experimental observations on their preceding nodes. For both (PIK3R.) and Cell Migration nodes, the observed variation cannot be deduced from variations in their precursors (PDGFRB for (PIK3R.), see Figure 3A, and (RAC.) and (RHO.) for Cell Migration, Figure 3B). This suggests that the observed inconsistencies are due to deficiency in the network topology representation: the experimental observations were made at mRNA level (microarray data), but they are associated with nodes that represent proteins (interactions of the inconsistent parts are post-translational). This supports our proposition of differentiation between transcriptional and post-translational products in regulatory networks.

The original network was then modified by separating mRNAs and proteins as described in the Method section (Additional file 2: Modified interaction graph). BioQuali analysis of the new enriched interaction network with the observation dataset showed that the new network is consistent with the experimental observations.

### Predictions on the enriched network

The analysis of the enriched network using BioQuali resulted in 31 predictions about variations corresponding to different node types, as follows: 4 predictions made about mRNAs, 17 predictions made about proteins (with one protein representing a phosphorylated protein), 5 predictions made about nodes representing protein complexes and 5 predictions made about nodes representing protein groups/families (Table 2). All variations predicted by BioQuali - except those about protein complex and phenotypic nodes - were compared to experimental observations on the expression levels of their corresponding mRNAs, according to the classification of predictions introduced in the Method section. This classification identified eight predictions which allow us to discuss the existence of active regulations. Five predictions are of Type II - CDC2, IGF2, RB1-phosphorylated, TGFBR2 and TP73- and three predictions are of Type I for proteins or families -(RAC.), IGF1 and IGFBP3. The remaining 14 predictions on proteins and protein groups/families are of Type III.

**Table 2:** Summary of EWS-FLI1 network analysis

**Prediction**	**Product**	**Deduction type**	**Functional type**	**Source of the prediction**
**Protein nodes**				

(PIK3C.) = +		Forward	Type III	obs. mRNA (PIK3C.), pred. (.RAS), pred. IGF1
(.RAS) = +		Backward	Type III	pred. IGFBP3, obs. mRNA IGFBP3
(RAC.) = -	Group of proteins	Backward	Type I	obs. mRNA (RHO.) and obs. cell migration
(TGFB.) = +		Forward	Type III	obs. mRNA TGFB
(TNF.) = +		Forward	Type III	obs. mRNA TGFB

RB1_p = -	Phosphorylated protein	Forward	Type II	pred. PRKCB1

CDC2 = -		Forward	Type II	pred. (((CCNA.)_p):CDC2), pred. (CCNB1 p:CDC2)
ECM1 = +		Forward	Type III	obs. mRNA ECM1
ECM2 = +		Forward	Type III	obs. mRNA ECM2
FASLG = +		Forward	Type III	obs. mRNA FASLG
IER3 = +		Forward	Type III	obs. mRNA IER3
IGF1 = +		Backward	Type I	obs. mRNA ACT1
IGF2 = -		Backward	Type II	obs. mRNA CDKN1C, pred. TP73
IGFBP3 = -	Proteins	Backward	Type I	pred. IGF1, obs. mRNA IGF1
JUN = +		Backward	Type III	obs. mRNA FASLG
MYCBP = -		Forward	Type III	obs. mRNA MYCBP
PRKCB1 = -		Forward	Type III	obs. mRNA PRKCB1
RASA1 = +		Forward	Type III	obs. mRNA RASA1
SKP2 = +		Forward	Type III	obs. mRNA SKP2
TGFBR2 = +		Forward	Type II	pred. mRNA TGFBR2
TNFAIP3 = +		Forward	Type III	obs. mRNA TNFAIP3
TP73 = -		Backward	Type II	obs. mRNA PDGFRB

mRNA and protein complex nodes				

mRNA_BCL2 = +		Forward	-	pred. IGFBP3
mRNA_IGF2 = -	mRNAs	Backward	-	pred. IGF2, pred. IGFBP3
mRNA_TGFBR2 = +		Forward	-	obs. EWS-FLI1
mRNA_TP53 = -		Forward	-	pred. JUN

((CCNA.):CDK2) = -		Backward	-	obs. cell-cycle G2
((CCNA.)_p:CDC2) = -		Forward	-	obs. cell-cyle M
((CCNA.)_p:CDK2) = -	Protein complexes	Forward	-	obs. cell-cycle M
(CCNB1:CDC2) = -		Backward	-	obs. cell-cycle M
(CCNB1_p:CDC2) = -		Forward	-	obs. cell-clycle anaphase

List of predictions deduced from EWS-FLI1 regulatory network. 'Deduction' corresponds to the direction of propagation of the regulation in the network. The first part of the table corresponds to functional predictions about proteins. The 'Functional type' column shows the result of the post-processing classification of these predictions detailed in the Method section. The second part of the table gathers relevant predictions about other nodes in the network, that is mRNA and protein-complexes. Although they do allow discussing the dogma of a correlation between mRNA and protein levels, such predictions are used in the Results section to discuss the relevance of predictions about proteins. For each prediction we indicate the source of prediction, which is the minimal set of nodes (observed or predicted) that is needed for the deduction of the prediction. For instance, the first line reads as follows: the prediction about (PIK3C.) protein node is a forward deduction from three sources: the observation on (PIK3C.) mRNA and the predictions about (.RAS) and IGF1 nodes.

**Figure 4:**
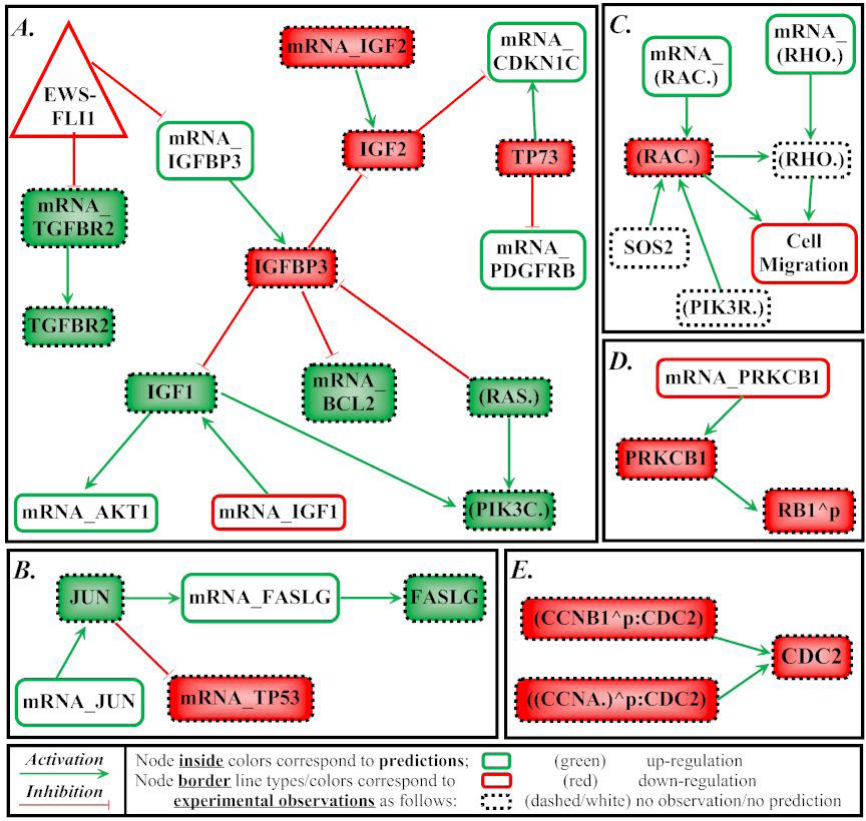
**Representation of predictions in the network**. This figure shows parts of the network where the predictions considered in the section 'Discussion' are located. It helps backtracking the source of each prediction. *Part A*. Predictions on TGFBR2 mRNA and protein are deduced from the observation on EWS-FLI1. The prediction about BCL2 mRNA is deduced from the prediction about IGFBP3, deduced from the prediction about the IGF1 protein which, in turn, is deduced from the observation on AKT1 mRNA. The prediction about the TP73 protein is deduced from the observation on PDGFRB mRNA and serves, together with the observation on CDKN1C mRNA, as a source of the prediction about the IGF2 protein and subsequently - about IGF2 mRNA. The prediction about the family of proteins (.RAS) is deduced from the prediction about the IGFBP3 protein and, together with the prediction about the IGF1 protein, is a source of the prediction on family of proteins (PIK3C.). *Part B*. Prediction about the JUN protein is deduced from the observation on JUN mRNA and serves as a source for the prediction about the TP53 mRNA. Prediction about the FASLG is a forward deduction from the observation on the FASLG mRNA. *Part C*. Prediction about the (RAC.) family of proteins is deduced as the only possible explanation of the combination of observations on (RHO.) mRNA family and the phenotypic node [Cell Migration]. *Part D*. Prediction on phosphorylation of RB1 is a forward deduction from the prediction on PRKCB1 which is deduced from the observation on PRKCB1 mRNA. *Part E*. The prediction about the CDC2 protein is a result of the correlated interactions of its precursors, predictions on complexes ((CCNA.)^ p:CDC2)) and (CCNB1^ p:CDC2).

**Figure 5:**
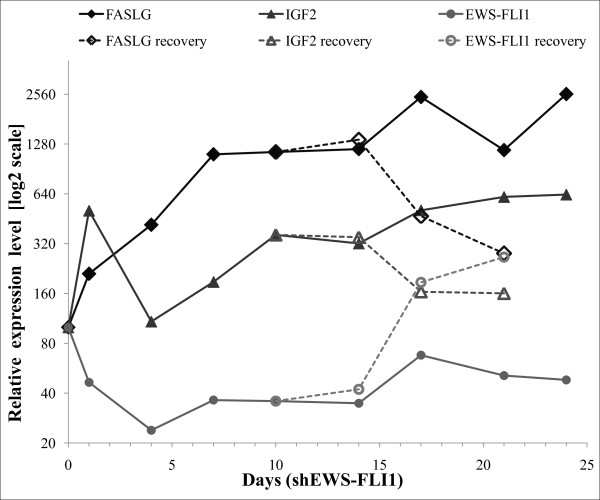
**RT-QPCR data on FASLG, IGF2 and EWS-FLI1 time series**. Quantification of FASLG, IGF2 and EWS-FLI1 was performed by RT-QPCR in a time series experiment. EWS-FLI1 was silenced upon shRNA induction by addition of doxycycline in the media for up to 24 days. For the recovery series, doxycycline was omitted from the media after the 10th day of culture (dashed lines).

**Figure 6:**
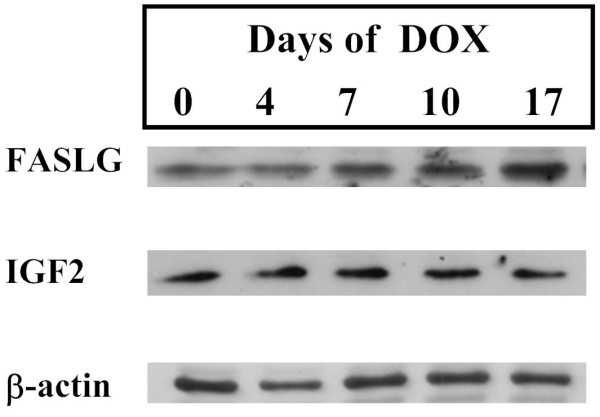
**Western blotting on IGF2 and FASLG**. Western blotting of FASLG, IGF2 and beta-actin in a time series experiment. EWS-FLI1 was silenced upon shRNA induction by addition of doxycyclin in the media for up to 17 days.

From the 14 predictions of Type III, a path of predictions was selected for experimental validation of the approach. We considered FASLG as an important node to be checked since the BioQuali logics applied to its transcript variations both implies that the protein FASLG is increasing and that JUN, the only precursor to FASLG mRNA, is increasing as well - see Figure 4B for details. Results of quantitative real-time PCR confirmed that FASLG mRNA anti-correlates with EWS-FLI1 (Figure 5), inducing an increasing variation of the transcript during the oncogene inhibition. Western blot on FASLG protein (Figure 6) confirms the 'forward' prediction 'FASLG = '+".

### Type II predictions

Type II predictions are more informative than those of Type III since they correspond to proteins which mRNAs were not observed with a significant *p*-value during the oncogene inhibition. This suggests that a phenomenon occurs at the post-translational level which is not initiated at the transcriptional level.

The prediction about the TGFBR2 protein is a direct consequence of another prediction, about its mRNA. Indeed, TGFBR2 mRNA is predicted as '+' by a forward prediction sourced in its inhibition by the inhibited oncogene EWS-FLI1, its only precursor (Figure 4A and Table 2). The production of TGFBR2 is regulated by its transcript only, and is therefore activated as well. This is in agreement with previously reported down-regulation of TGFBR2 by EWS-FLI1 [26,21,27]. This also points out a lack in sensitivity of the Affymetrix data, which did not report a significant variation of TGFBR2 mRNA.

Among the other Type II predictions, we would point out the prediction about the protein TP73 as down-regulated during the inhibition of EWS-FLI1. The source of the prediction is the following: TP73 is the only inhibitor of PDGFRB mRNA which is observed as up-regulated (Figure 4A). This backward deduction suggests that one of the incoming regulations of TP73 protein (activations by TP73 mRNA, MAPK1 and EP300 and inhibitions by MDM2 and MYC, not shown) may be activated by the inhibition of the fusion oncogene. This prediction about TP73 is of great importance since it will result in a new prediction about IGF2 and its mRNA and will finally allow us to propose network refinements.

### Type I predictions

Our post-processing of BioQuali output pointed out three Type I predictions, which directly indicate active post-translational regulations in the network. These are predictions about IGF1, IGFBP3 and the (RAC.) family of proteins which includes RAC1, RAC2 and RAC3.

#### (RAC.) case study

The prediction about (RAC) node results from a path of reasoning in several steps. We refer to Figure 4C for details. The prediction is a backward deduction from the observation on the phenotypic node [Cell Migration] ([cell migration] = '-'). This phenotypic node has two precursors: (RAC.) and (RHO.). Notice that (RHO.) also has two precursors: its mRNA (observed as "+") and the node (RAC.). According to the logic of BioQuali, the observed variation of [Cell Migration] must be explained by one of the incoming regulations, which means that one of the two precursors must have a variation '-'. (RAC.) being set as '+' would imply that (RHO.) receives two positive influences, which would finally imply that [Cell Migration] is positive, contradicting the observation on this node. This makes the original hypothesis ((RAC.) = '+') impossible and therefore (RAC.) is predicted as '-'.

However, the input dataset showed that the transcript variation of the (RAC.) family is clearly positive. This suggests that one of the post-translational regulations on (RAC.) protein (in our network they come from SOS2 protein and (PIK3R.) family) is active and reverses the effect of the production of RAC transcripts.

#### IGF1 and IGFBP3 case study

The predictions about IGF1 and IGFBP3 will be discussed together since they are deeply connected.

The prediction about the IGF1 node is a backward deduction from the observation on AKT1 mRNA (Figure 4A). The increase of IGF1 protein is the only possible explanation of its target - AKT1 mRNA - up-regulation. The IGF1 node is the target of two incoming regulations: from its transcript and from IGFBP3 protein. However, the transcript is observed as significantly decreasing in the Affymetrix chip. This suggests that the predicted behavior of IGF1 protein can only be explained by the inhibition issued in the IGFBP3 protein. The effect of IGFBP3 competes with the protein production from its transcripts and eventually reverses it.

The predicted variation on IGFBP3 protein is also of the backward type. It is a direct consequence of the variation of IGF1 protein: since IGFBP3 is the only inhibitor of IGF1, and since IGF1 must increase from the previous analysis, we deduce that IGFBP3 protein is decreasing during the experiment. Nonetheless, its transcripts increase significantly during the experiment. This suggests that the production of IGFPB3 results from a competition between the translation of its transcript and the regulation from the (RAS.) pathway. Here, the "competition" is won by (RAS.).

This prediction was compared with the previously reported data shown in Figure 6A of [28]. A discrepancy appeared since these new data suggest that IGFBP3 protein is induced upon silencing of EWS-FLI1.

### Towards a network refinement

#### Using predictions to propose a relevant set of additional observations

In order to explain the discrepancy between the prediction about IGFBP3 (deduced from the network topology) and the previously reported observation about this protein [28], a new range of experiments was designed. We pointed out the IGF2 node in the network since it involves a prediction of Type II being is a direct consequence of several predictions discussed above.

Indeed, the prediction about IGF2 protein is a three-step backward deduction. It is due to the combination of observations on CDKN1C mRNA and PDGFRB mRNA (Figure 4). The observed variation of CDKN1C mRNA is '+', and it has two incoming interactions: from proteins TP73 and IGF2. According to BioQuali, the observation on CDKN1C mRNA must be explained by one of these entries. It cannot be explained by regulation from the TP73 protein (prediction addressed in details above). Therefore, the only possible explanation of the observed variation of CDKN1C mRNA is its inhibition by IGF2. In this case, the variation of the IGF2 protein must be '-', as it is stated in the prediction listing (see Table 2 and Additional file 5: Results of the constraint-based analysis). Pushing the reasoning further, the predicted variation of IGF2 protein must be explained by one of its incoming interactions, either by the inhibition from the IGFBP3 protein or by the activation from IGF2 mRNA (Figure 4A). According to the prediction about IGFBP3 protein, IGF2 mRNA can be the only explanation of predicted variation of IGF2 protein (prediction: IGF2 mRNA = '-').

In other words, according to the reasoning we have used up to now, both IGF2 and its mRNA should decrease during the oncogene inhibition, and this behavior is shown to be characteristic from the network topology and Affymetrix observations. Both these variations were checked experimentally. RT QPCR showed that the level of IGF2 mRNA increases during the inhibition of EWS-FLI1 (Figure 5) and Western blotting showed that IGF2 protein does not respond significantly to the inhibition of EWS-FLI1 (Figure 6). As for observation from [28], this was in complete disagreement with the variations deduced from the topology of the model.

#### Re-performing the analysis and pointing out lacks in the network

A new set of observations was built according to the new data at hand: the significant variations observed in the Affymetrix chip were complemented by two additional data: 'IGF2_mRNA = +' and 'IGFBP3 = +'. The BioQuali analysis of the network with respect to this set of variations resulted in an inconsistent diagnosis, indicating that regulations are missing over IGF1 to explain the new set of observations. This points out the need for further biological investigations of this pathway to complete the picture of regulations and pathway cross-talks in Ewing's cancer.

This situation illustrates the utility of our approach, which points out weak parts of the model that should be submitted for thorough experimentation. The conflicts discovered also suggest those parts of the network model (a priori generic) which should be adapted to the specificities of the cell line and phenotype. In this case, our theoretical investigations of the EWS-FLI1 regulatory network suggest that the IGF pathway could be an important factor in the development of Ewing's cancer. This agrees with previously reported Identification of IGF1 and IGFBP3 as EWS-FLI1 target genes in [28-31].

## Conclusion

We have used a network representation to model the behavior of genes controlled by an oncogene in the context of Ewing's sarcoma. The network gathers "gold standard" but generic information on pathways involved in cell proliferation and apoptosis, which are putatively under the influence of the oncogene. The network has been modified to include post-translational interactions and to contain both mRNA and protein nodes. The analysis allowed us to pinpoint active interactions specific to this cancer. We have also identified those parts of the network which were incomplete and should be submitted for further investigation.

The analysis was performed using the open-source software tool BioQuali and consisted of an automatic constraint-based analysis mimicking biologist's local reasoning on a large scale. The logical constraints used by the tool were proved to be relevant for our experimental setting - steady state shifts - in a previous mathematical work [24].

The results from the tool were post-processed by (1) differentiating between transcriptional and post-translational products of gene expression (2) analyzing the predicted variations to localize potentially active regulations and suggest network corrections. The underlying idea behind this process was that relevant information will be derived from situations where the naive application of the transcription dogma in eukaryotes could not apply.

The originality of our regulatory network analysis lies in the combination of prediction and localization of inconsistencies and contradictions. Predictions enhance the set of observable nodes by including products that are not accessible to experiments (such as active proteins in transcriptomic studies) or for which the accuracy of measurement is insufficient. An enhanced network can render complementary analysis such as sub-network classification methods and hypothesis generation more efficient. It also pinpoints active interactions and reveals disease specificities. Network failures and inconsistencies such as missing interactions or errors in node interpretation can also be localized in our approach. Corrections and further experiments are proposed in this case.

Last but not least, this type of analysis is iterative. As with any predictive method in systems biology, the results of the consistency checking depend on both the network and experimental data at hand: an inconsistency diagnosis locates lacunae in the model with respect to available data; predictions are direct consequences of those available data. Therefore, switching to another dataset may considerably modify the set of predictions and even change the result of the consistency checking if the network topology fails to explain the new set of inputs. Concretely, predictions in a first step can become inconsistencies when confronted with new data and require network correction in further steps. This happened in present work: applying the method to Ewing's sarcoma network and data we suggest that the full dataset explains a few post-translational processes. The post-processing of the results of the analysis led to proposition of the complementary experiments. These complementary experiments validated some of the predictions but also revealed lacks in the generic network. We have confirmed the specific role of IGF in the development of Ewing's cancer and have localized parts of this pathway that should be studied as a priority in the future.

More generally, it is crucial to build networks in a manner that realistically takes into account various sources of expression variation (transcription factors activities, alternative splicing, post-translational modifications of proteins, etc.). Thus, other elements shall be considered in a network structure, and extensions are in progress. These may be microRNAs, mRNAs, proteins, phosphorylated proteins, etc. depending on the type of the experimental data available to the modeler. For instance, our formalism can easily integrate the effect of genetic amplifications by introducing DNA node type whose attribute is the gene copy number variation (work in progress). Alternative splicing is simply a question of multiplying the proteins nodes resulting from a given mRNA. Our approach is a first step forward in this direction.

## Authors' contributions

TB conceived the study and performed the analysis. DS and OD produced experimental data for predictions validation. TB, BD and OR performed statistical analysis of microarray data. GS built the regulatory network, further adjusted by TB for this study. TB, OR and AS wrote the manuscript. AS guided the project. All authors read, corrected and approved the final manuscript.

## Supplementary Material

Additional file 1 **Interaction graph adapted from original network**. The non-modified interaction graph (no differentiation between mRNAs and proteins done) is shown in the file 'Additional file 1.csv', which is structured to be readable by the BioQuali tool. Click here for file

Additional file 2 **Modified interaction graph**. 'Additional file 2.cvs' contains the network which includes all modifications as described in the text of the paper. It is also in a BioQuali-readable format of interaction network representation. Click here for file

Additional file 3 **Results of statistical analysis of microarray data **[21]. 'Additional file 3.xls' comprises calculated slopes, variations and corresponding p-values for microarray data of gene responses on both inhibition and recovery of EWS-FLI1. All the transcript variants of genes present in our network model were grouped and the statistical tests were applied to such 'gene' data rather than 'transcript variant' data, to give a comprehensive picture of gene behaviors. Click here for file

Additional file 4 **Set of experimental observations**. 'Additional file 4.csv' file presents the observation dataset resulting from the statistical analysis of microarray data shown in Additional file 3. Click here for file

Additional file 5 **Results of the constraint-based analysis**. 'Additional file 5.xls' contains the results of constraint-based modeling of the extended EWS-FLI1 regulatory network with the integrated experimental observations set. The first column shows the discovered inconsistencies (while analyzing the original interaction network). The following columns display the consistency of the modified network (mRNA nodes included) with the dataset and predictions obtained after the analysis. Click here for file
